# Angiogenic Potential of Vitreous from Proliferative Diabetic Retinopathy and Eales' Disease Patients

**DOI:** 10.1371/journal.pone.0107551

**Published:** 2014-10-13

**Authors:** Ponnalagu Murugeswari, Dhananjay Shukla, Ramasamy Kim, Perumalsamy Namperumalsamy, Alan W. Stitt, Veerappan Muthukkaruppan

**Affiliations:** 1 Department of Immunology and Cell Biology, Aravind Medical Research Foundation, Dr.G.Venkataswamy Eye Research Institute, Madurai, India; 2 Vitreous and Retina Service, Aravind Eye Care System, Madurai, India; 3 Centre for Experimental Medicine, Queens University, Belfast, United Kingdom; Medical University Innsbruck, Austria

## Abstract

**Purpose:**

Proliferative Diabetic Retinopathy (PDR) and Eales' Disease (ED) have different aetiologies although they share certain common clinical symptoms including pre-retinal neovascularization. Since there is a need to understand if the shared end-stage angiogenic pathology of PDR and ED is driven by common stimulating factors, we have studied the cytokines contained in vitreous from both patient groups and analyzed the angiogenic potential of these samples *in vitro*.

**Material and Methods:**

Vitreous samples from patients with PDR (n = 13) and ED (n = 5) were quantified for various cytokines using a cytokine biochip array and sandwich ELISA. An additional group of patients (n = 5) with macular hole (MH) was also studied for comparison. To determine the angiogenic potential of these vitreous samples, they were analyzed for their ability to induce tubulogenesis in human microvascular endothelial cells. Further, the effect of anti-VEGF (Ranibizumab) and anti-IL-6 antibodies were studied on vitreous-mediated vascular tube formation.

**Results:**

Elevated levels of IL-6, IL-8, MCP-1 and VEGF were observed in vitreous of both PDR and ED when compared to MH. PDR and ED vitreous induced greater levels of endothelial cell tube formation compared to controls without vitreous (P<0.05). When VEGF in vitreous was neutralized by clinically-relevant concentrations of Ranibizumab, tube length was reduced significantly in 5 of 6 PDR and 3 of 5 ED samples. Moreover, when treated with IL-6 neutralizing antibody, apparent reduction (71.4%) was observed in PDR vitreous samples.

**Conclusions:**

We have demonstrated that vitreous specimens from PDR and ED patients share common elevations of pro-inflammatory and pro-angiogenic cytokines. This suggests that common cytokine profiles link these two conditions.

## Introduction

Proliferative Diabetic Retinopathy (PDR) and Eales' Disease (ED) are potentially blinding vitreoretinal diseases. ED is an idiopathic inflammatory venous occlusion that primarily affects the peripheral retina of healthy young men (20–30 years). Retinal changes in ED include periphlebitis, peripheral non-perfusion and neovascularization. In addition, visual loss is characteristically caused by bilateral recurrent vitreous hemorrhage [1], [2]. Although PDR and ED have different etiology, the symptoms and signs of these diseases run parallel to each other with some differences [3]. We have previously observed this commonality in the presence of inflammatory cytokines [interleukin-6 (IL-6), interleukin-8 (IL-8), monocyte chemoattractant protein-1 (MCP-1)] and vascular endothelial growth factor (VEGF) in vitreous [4].

Angiogenesis is a highly complex and coordinated process requiring multiple receptors and ligands in endothelial cells for which VEGF is a pivotal element in a variety of normal and pathological circumstances [5], [6]. VEGF is upregulated during retinal hypoxia and levels of this growth factor are raised in the vitreous of patients with PDR [7], [8] and VEGF-neutralizing antibodies are now being used for treatment of pathological ocular angiogenesis and macular oedema [9], [10], [11], [12].

Though the presence of pro-angiogenic and pro-inflammatory cytokines in the vitreous of PDR and ED patients has been demonstrated by several studies the functional potential of their vitreous has never been shown. Therefore, we have investigated the angiogenic potential of vitreous from these patients using the endothelial tube formation assay *in vitro*, and the effects of anti-VEGF and anti-IL6 neutralizing antibodies on this potential.

## Materials and Methods

### Ethics Statement

Patients were recruited in accordance with the Declaration of Helsinki and with the approval of the Institutional Review Board (IRB) of Aravind Eye Hospital. Written informed consent as per the procedure approved by IRB was obtained from the patients and maintained.

### Study Subjects

Consecutive patients scheduled to undergo vitrectomy for proliferative ED, advanced PDR or Macular Hole (MH) were prospectively recruited in one of the three treatment groups. The clinical diagnosis of ED was made on the basis of peripheral venous sheathing in multiple quadrants in the fellow eye, or the operated eye, as observed intra-operatively. The patients with conditions which could secondarily cause venous sheathing, such as non-inflammatory retinal vein occlusions, diabetic or hypertensive retinopathy, sickle cell retinopathy, ocular inflammatory conditions like choroiditis, or pars planitis, or associated systemic infective/autoimmune/other inflammatory disease (as revealed by history, examination, or investigations), were excluded from the study. The indications for vitrectomy included best-corrected visual acuity less than 20/400 due to vitreous haemorrhage of at least 2 months duration with/without epiretinal membranes; rhegmatogenous/combined-mechanism retinal detachment; or a tractional retinal detachment involving the macula. Eventhough, haemorrhage is an important indication for vitrectomy; small samples of undiluted vitreous were obtained before isotonic infusion and aspiration. The samples which contained blood contamination were excluded from the study.

All the diabetic patients included in this study had type 2 diabetes mellitus. Like ED patients, they were also surgical inpatients, who presented with advanced PDR, as defined by the ETDRS study report number 12 [13]. The indications for vitrectomy were the same as mentioned above for ED patients.

Patients with idiopathic MH were chosen as control. MH is caused by vitreomacular traction, without any associated retinal ischaemia, vascular proliferation or inflammation, and is therefore least likely to be associated with local release of VEGF or other inflammatory cytokines. MH patients were evaluated to rule out a history or signs of ocular trauma or any other associated ocular pathology. The main preoperative investigation in MH was optical coherence tomography, to assess the morphology of MH for surgical feasibility and prognosis. MH patients had no systemic diabetes mellitus. Patients were recruited in accordance with the Declaration of Helsinki and with the approval of the Institutional Review Board of Aravind Eye Hospital. Informed consent was also obtained from the patients. All the cases had no history of a previous ocular surgery.

Thirteen PDR patients (11 males, 2 females), aged 54.2±7.8 years (mean ± SD) having diabetes for 13.0±9.7 years were included in the study. All 5 ED patients were males and 31±10 years old. Five MH patients (1 female, 4 males) aged 60.4±8.2 were included as controls. All the PDR and ED patients had retinal neovascularization and vitreous haemorrhage. As the etiology is different [14], the ED patients were males and relatively younger in age. Therefore, this difference in age and sex is admissible.

### Sample Collection

At the beginning of vitrectomy, undiluted vitreous (200–700 µl) was aspirated via pars plana with a vitreous cutter, before opening the infusion port. These undiluted vitreous samples (13 PDR, 5 ED and 5 MH) were immediately frozen in aliquots in polypropylene tube at −80°C until assay.

### Cytokine Assay

Vitreous samples (PDR, n = 8; ED, n = 2) were quantified for a range of cytokines (IL-2, IL-4, IL-6, IL-8, IL-10, IL-1α, IL-1β, IFN-γ, VEGF, TNF-α, MCP-1, EGF) using the Evidence Investigator Cytokine Biochip Array (Randox, UK). For each sample, this assay was carried out in duplicate. It is a biochip with multiple cytokine antibodies coated on a solid substrate. After incubation with vitreous samples, cytokine specific enzyme-labelled secondary antibodies were added, and the cytokines were quantified by chemiluminescence using a charged coupled device (CCD) camera. The standard curves were constructed for each cytokine and the sample concentration was determined by the evidence investigator (Randox, UK). As a follow-up, IL-6, IL-8, MCP-1, and VEGF were also quantified by sandwich ELISA (BD Biosciences, R & D systems) according to the manufacturer's instructions for 5 PDR, 3 ED and 4 MH samples. The standard curve was prepared using recombinant human cytokines.

### Tubulogenesis assay

Human dermal microvascular endothelial cells (HMECs) were purchased (PromoCell Gmbh, Heidelberg, Germany) and grown in T-25 Nunclon culture flasks (Nalge Nunc International,UK) with medium (PromoCell) supplemented with growth supplements (PromoCell) and Primocin (50 mg/ml) (Invitrogen,UK). The *in vitro* tubulogenesis assay was performed as previously described using confluent cells of passage four to six [15]. Briefly for each assay 2×10^4^ HMECs in 5 µl were resuspended in 10 µl of vitreous and mixed with 15 µl of growth factor reduced matrigel (BD Biosciences,USA) (PDR, n = 6; ED, n = 5, MH n = 5). In place of vitreous 10 µl of medium was used in controls.

In another series of experiments, HMEC cultures were treated with ranibizumab (RZB) (0.125 mg/ml or 0.25 mg/ml; PDR, n = 6; ED, n = 5) (Genentech,Inc., South San Francisco) or anti-IL-6 (0.1 µg/ml; PDR,n = 7;ED,n = 2) neutralizing antibody (R & D systems, UK). IL-6 was chosen in preference to other cytokines and chemokines, since it is known to be elevated in vitreous and plays a role in the pathogenesis of ocular diseases [4], [16], [17]. Moreover, previous studies have demonstrated that IL-6 levels in aqueous and vitreous fluids from PDR patients significantly correlate with disease severity [17], [18], [19]. The above mixture of matrigel, HMEC, and RZB was prepared collectively for 3 assays (n = 3) and anti-IL-6 mixture for 2 assays for each sample. Thirty microliter aliquots from the mixture were spotted for each assay onto Nunclon 48-well culture plates. After matrigel polymerization at 37°C for 30 minutes, blobs were covered with endothelial cell supplement medium. Tube formation was observed after 48 hours, phase images were captured in five regions per well and tube length was quantified using NIS-Elements software (Nikon, UK). The mean tube length of all the five regions of triplicate/duplicate was obtained.

### Statistical Analysis

The Man-Whitney U test was used to analyse the differences between the experimental and control groups. Values are expressed as mean ± SD. All analysis was done using statistical software Stata 11.0. The results were considered significant at P<0.05.

## Results

### Cytokine Profile

**Figure 1:**
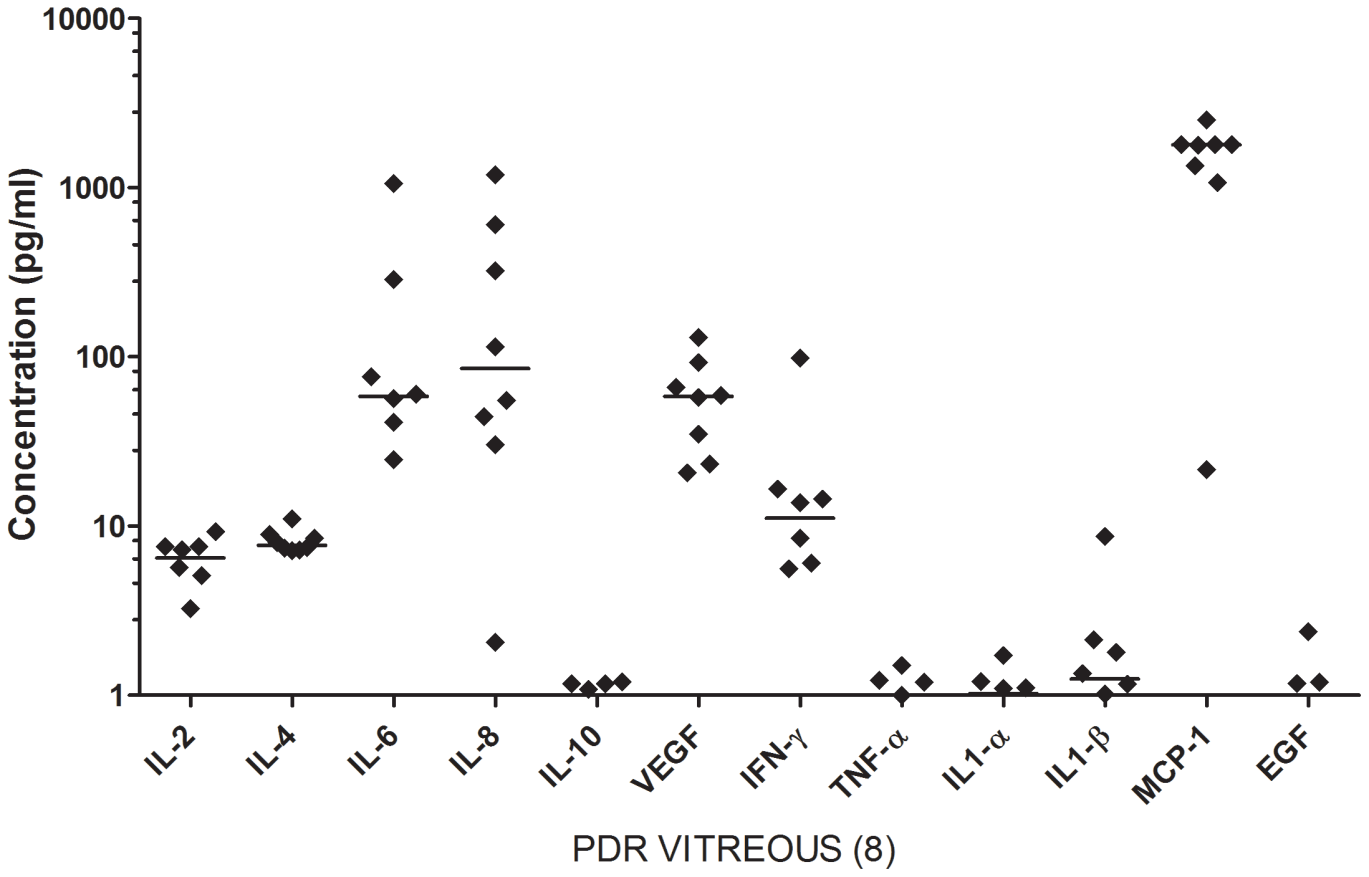
Scatter plot showing the distribution levels of 12 cytokines in vitreous from PDR (n = 8) patients, quantified using cytokine bio-chip array. Each sample represents the mean of duplicates. Solid line indicates the median. PDR-Proliferative diabetic retinopathy; ED- Eales' disease. IL - Interleukins, VEGF- Vascular endothelial growth factor, IFN-γ- Interferon gamma, TNF-α - Tumour necrosis factor alpha, MCP-1 - Monocyte chemoattractant protein-1, EGF - Epidermal growth factor.

We have earlier demonstrated that significantly higher concentrations of IL-6, IL-8, MCP-1 and VEGF were observed in vitreous of PDR (n = 25) and ED (n = 10) than in that of MH patients (n = 25) [4]. The above study also showed strikingly similar cytokine profile in both PDR and ED vitreous and this finding is further confirmed by an extremely rapid cytokine biochip array (Figure 1, Table 1). Moreover, only trace levels of IL-10, TNF-α, IL1-α, IL1-β and EGF were observed in both groups.

**Table 1:** Levels of cytokines in PDR, ED and MH vitreous used for tubulogenesis assay.

Sample Code	IL6 (pg/ml)	IL8 (pg/ml)	VEGF (pg/ml)	IL-1β (pg/ml)	MCP1 (pg/ml)
*PDR-27	0	0	849.2	-	978.2
* PDR-29	0	0	0	0	0
* PDR-38	260.2	38.0	2401.4	0	2486.9
* PDR-40	225.1	89.4	682.7	0	2571.0
* PDR-45	0	228.3	5805.7	0	1863.6
† PDR-49	>530	30.1	130.2	0	1349.0
† PDR-53	0	2.1	20.6	2.1	21.6
† PDR-58	60.3	604.2	57.5	1.7	>900
† PDR-61	56.6	55.4	66.0	1.0	>900
† PDR-66	24.6	44.3	59.1	0.6	1071.0
* ED-09	0	290.2	105.7	23.8	2063.0
* ED-12	175.0	632.6	0	2.0	-
* ED-16	-	148.1	221.2	3.2	-
† ED-17	38.5	56.9	144.0	0.3	1017.9
† ED-19	158.9	961.0	204.9	2.6	>900
* MH-09	0	4.9	0	103.7	118.4
* MH-29	0	0	0	35.1	937.8
* MH-35	0	0	0	0	0
* MH-38	0	0	107.8	0	174.0
MH-44	-	-	-	-	-

The cytokine levels were analysed by cytokine bio-chip array for 5 PDR, 2 ED vitreous (indicated by †) and the remaining (PDR  = 5, ED  = 3, MH  = 4) by sandwich ELISA (indicated by*). The values in the table represent the mean concentration duplicate of each sample. For the two ED (†) samples, cytokines not included in this table are: IL-2, 8.1 pg/ml; IL-4, 7.14 pg/ml; IFN- γ, 12.8 pg/ml and less than 1.6 pg/ml for IL-10, TNF-α, IL-1α. 0- Less than the detectable limit; - cytokine not analysed; > Higher than the maximum detectable limit. PDR - Proliferative diabetic retinopathy; ED - Eales' disease; MH - Macular Hole.

### Endothelial Tube Formation induced by patient vitreous

**Figure 2:**
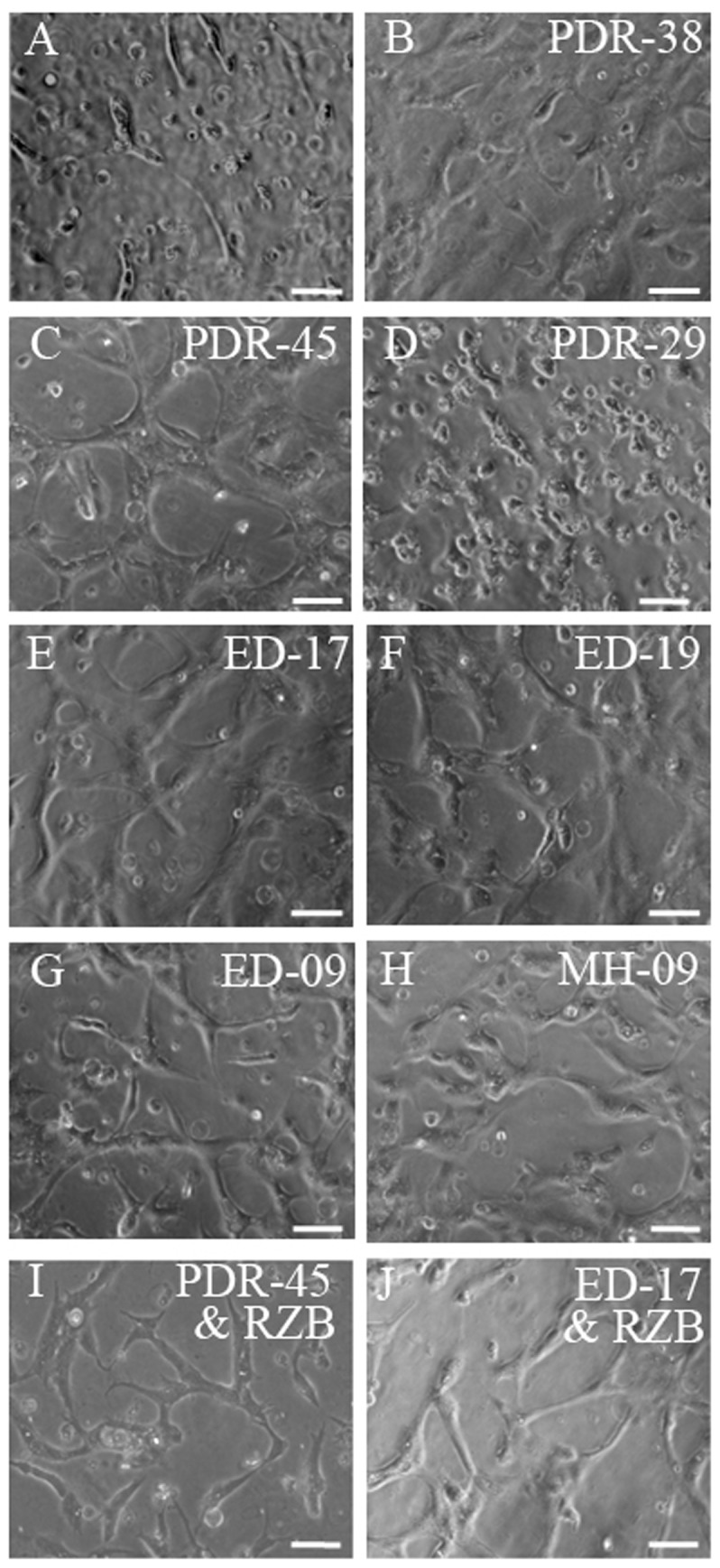
Representative phase images of tube formation induced by vitreous from PDR/ED/MH patients in human dermal microvascular endothelial cell (HMEC). 2×10^5^ HMECs in triplicate were exposed to vitreous alone or with RZB (0.125 mg/ml). Tube formation was observed and images were captured after 48 hours incubation. Each panel shows a part of the representative well. The tube length was quantified by NIS-Elements software (Nikon). Scale bar  = 100 µm. A. Control (without vitreous); B and C - PDR vitreous-induced tube formation, which had high levels of VEGF/IL-6/MCP-1; D-PDR vitreous with trace levels of cytokines showing a very few tube formation. E- G - ED vitreous-induced tube formation as in PDR. H - MH vitreous. I and J are images of vascular tubes in the presence of PDR/ED vitreous and anti-VEGF antibody, showing reduction in tube length compared to C and E respectively. Number in the images denotes the patient ID as in table 1. PDR - Proliferative diabetic retinopathy; ED- Eales' disease; MH- Macular Hole. RZB – Ranibizumab.

**Figure 3:**
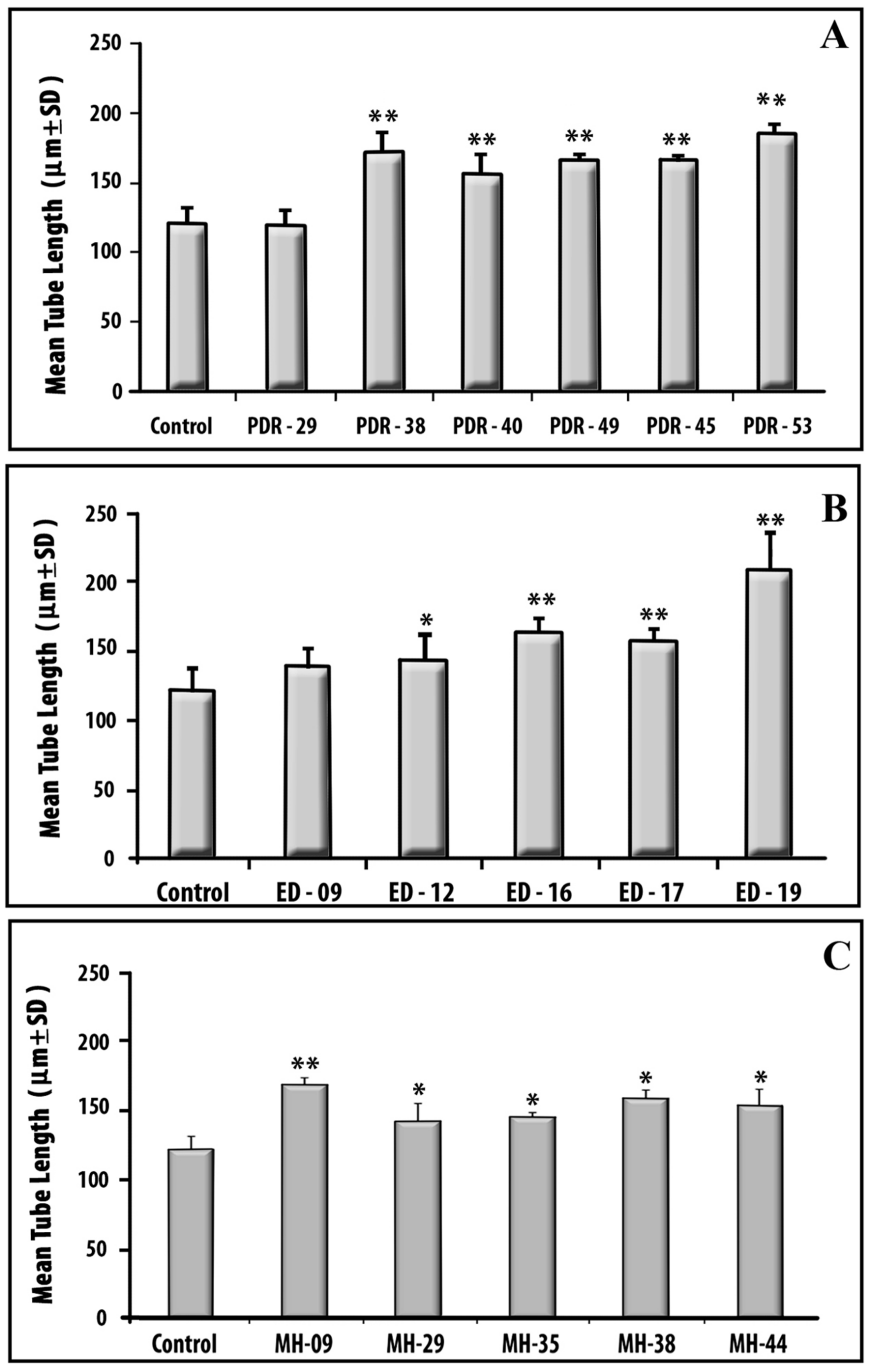
Angiogenic potential of vitreous in capillary tube formation. Experimental details are as in Fig. 2. (A) PDR vitreous, (B) ED vitreous, (C) MH vitreous. Bar graph shows the mean concentration of the triplicate of each sample. PDR-proliferative diabetic retinopathy; ED- Eales' disease; MH- Macular Hole. Number in X-axis denotes the patient number as in table 1. **P<0.001; *P<0.05.

The tubulogenesis assay is an *in vivo* correlate of angiogenesis involving endothelial alignment, elongation and polygonal network [20]. Cytokine concentrations estimated by ELISA or Biochip for PDR, ED and MH vitreous, which have been used for tubulogenesis assay, are presented in Table 1. After 48 hours of culture the endothelial cells in the absence of vitreous formed a few short tubes and majority of them remained as individual cells (Fig. 2A). Mean tubular length was 122 µm in control (Fig. 3). In some of the vitreous samples, there were more prominent polygonal tubule network and these had correspondingly high concentrations of VEGF or IL-6 (Fig. 2B and C). On the other hand, vitreous with only trace amounts of cytokines produced minimal tubule formation similar to the control (endothelial cells without vitreous) (Fig. 2D and 3A). Among the 6 PDR vitreous samples tested, 5 showed a significant increase in tube formation (mean fold increase ranging from 0.3 to 0.5, P<0.05, Fig. 3A).

Interestingly a similar vascular response pattern was observed with ED vitreous containing either high (ED-17 and 19; Fig. 2E and F) or low levels of (ED-09, Fig. 2G) cytokines (Table 1). Among, the 5 vitreous samples from ED patients 4 showed significant increase in tube formation (mean fold increase ranging from 0.1 to 0.7) (P<0.05; Fig. 3B).

In MH patients though the retinal vascular reaction does not occur in vivo, we observed significant tube formation with vitreous from all these patients (Fig. 3C). The nature of the vascular assembly is shown in figure 2H for vitreous of MH-09 which contained substantial amount of MCP-1 and IL1-β and trace level of VEGF (Table-1). With limited number of samples studied, it was not possible to apply a test for correlation. However, the data indicate that tube formation in vitro is influenced by the levels of various pro-angiogenic factors present in the vitreous samples.

### Effect of RZB on vitreous-induced Endothelial Tube Formation

**Figure 4:**
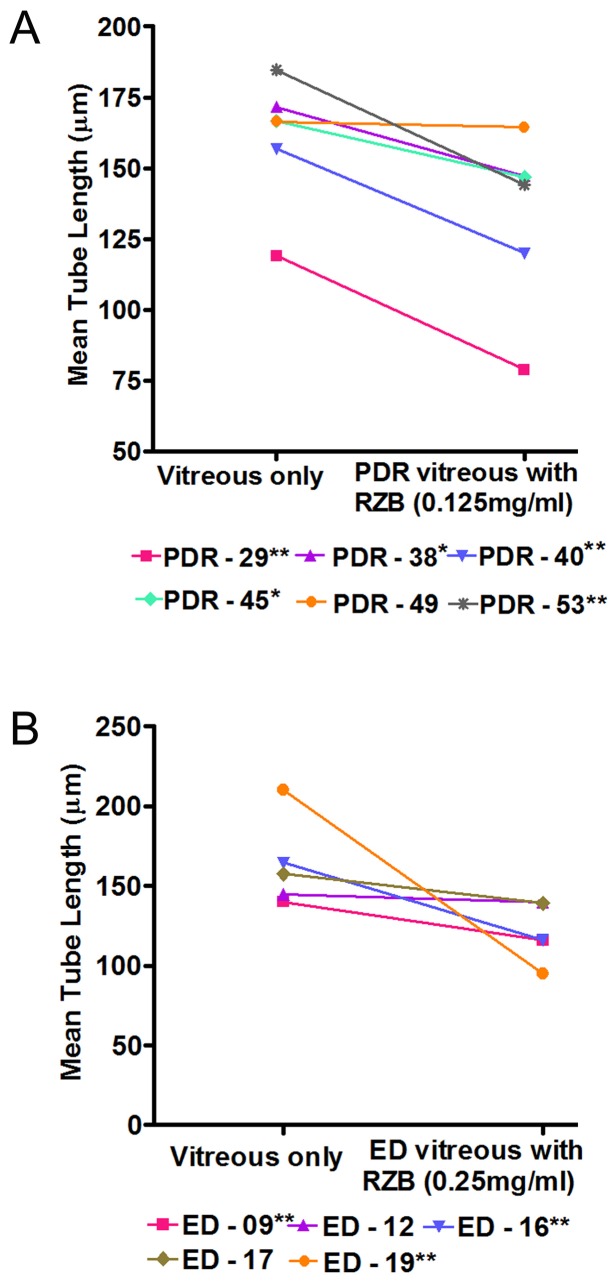
Effect of anti-VEGF antibody on vitreous-induced tube formation. HMECs were mixed with PDR vitreous in presence of RZB (in triplicate cultures). Tube formation was observed and images were captured after 48 hours incubation. (A) 0.125 mg/ml of RZB with PDR vitreous (B) 0.25 mg/ml of RZB with ED vitreous. The tube length was quantified by Nikon NIS-Elements software. Number in the legend denotes the patient ID as in table 1. PDR - Proliferative diabetic retinopathy; ED- Eales' disease; RZB-Ranibizumab. **P<0.001;*P<0.05.

HMECs were exposed to clinically-relevant concentrations of RZB in combination with the each of the vitreous samples from PDR and ED patients. Tube formation was observed and images were captured after 48 hours of incubation. Figures 2C and 2I show the nature of the vascular assembly in the presence or absence of RZB with PDR-45 vitreous. The tube length was reduced in all six cases with 0.125 mg/ml RZB and the mean fold decrease ranged from 0.2 to 0.7 though significantly in 5 cases (Fig. 4A). Interestingly, we have also observed the reduction in tube length when ED vitreous was treated with RZB (Fig. 2E and 2J). This anti-VEGF effect was observed in all five cases with a mean fold decrease ranging from 0.2 to 1.2, though showing a significant decrease in three cases (Fig. 4B).

### Effect of IL-6 neutralizing antibody on vitreous-induced endothelial tube formation

**Figure 5:**
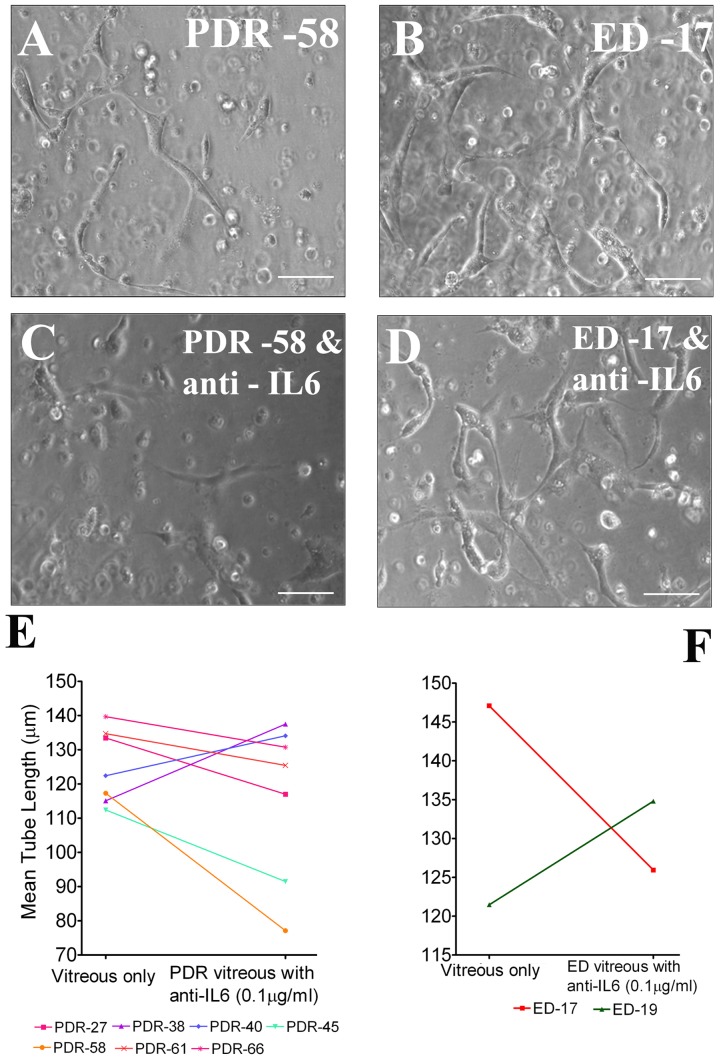
Effects of anti-IL-6 on vitreous-induced tube formation. HMECs were mixed with PDR or ED vitreous with or without anti-IL6 (0.1 µg/ml). Tube formation was observed and images were captured after 48 hours of incubation (in duplicate cultures). A and B are PDR or ED vitreous-induced tube formation. C and D are images of vascular tubes of corresponding vitreous with anti-IL6. E and F – shows the effect of anti-IL6 on vascular tube length for 6 PDR (E) and 2 ED (F) vitreous samples. The tube length was quantified by NIS-Elements software (Nikon). Number in the image denotes the patient ID as in table 1. PDR-Proliferative diabetic retinopathy; ED- Eales' disease. Scale bar  = 100 µm.

Since IL-6 was observed at high levels in PDR as well as in ED vitreous, we carried out another set of experiments to test the effect of anti-IL6 neutralizing antibody (0.1 µg/ml) on tube formation. Among seven samples of PDR vitreous tested, tube length was reduced in 5 samples with a mean fold ranging from 0.06 to 0.3 (Fig. 5A, C and E). Even though a noticeable reduction was observed in tube length, there was no significant difference between the groups with anti-IL-6 treatment. Our study included only two ED samples for anti-IL-6 treatment due to its reduced availability of clinical samples and limited volume of vitreous; of the two an apparent reduction in tube length was observed in one sample (ED -17) (Fig. 5B, D and F).

## Discussion

Angiogenic growth factors contribute to neovascularization that occurs in retinal diseases like DR and ED. In these vitreo-retinal diseases inflammatory processes are also considered to be critical, suggesting that a range of secreted factors in the vitreous cavity are associated with pathological processes [21], [22], [23]. The presence of inflammatory and angiogenic growth factors has been demonstrated in vitreous [4], [16], [24] from these patients. Though PDR & ED differ in their etiology the profile of proangiogenic factors in the vitreous is markedly similar. We have previously found no significant difference in the concentration of VEGF, IL-6, IL-8 and MCP-1 between these two diseases [4]. Moreover, the profile of twelve cytokines is also similar as shown by cytokine array (Fig. 1, Table 1) and this correlates with the other assays. This bio-chip approach is rapid and accurate taking <4 hours for analysis. It is known that some patients fail to respond to RZB therapy and in the future there may be a place for rapid vitreous analysis post-vitrectomy and establishing if certain growth factors or cytokines are absent or elevated. This would enable patient-specific tailoring the subsequent therapy.

Though there have been several reports on the presence of cytokines in vitreous of patients with vitreo-retinal diseases, to our knowledge, this is the first study to evaluate the angiogenic potential of vitreous from these patients. Using the *in vitro* tubulogenesis assay, we demonstrated the ability of PDR, ED, MH vitreous in inducing endothelial tube formation. In general, markedly increased number of vascular tubes and tubular network was observed with vitreous from the above patient groups than the control without vitreous. It is possible that the factors particularly VEGF, IL-6, IL-8 and MCP-1 might be responsible for vascular tube formation (Table 1, Fig. 3). Further, in addition to VEGF which is known to play a significant role in neovascularization in PDR [25], vitreous containing high levels of IL-6 and IL-8 or MCP-1 but with trace amount of VEGF also induced network of vascular tubes. (Table 1; Fig. 3; PDR-40, 49; ED-12, MH-09, 29). Therefore, in addition to VEGF, inflammatory cytokines are involved in inducing neovascularization.

There is substantial supporting evidence to indicate the interrelationship between the expression of inflammatory cytokines and vascular growth factors in different types of clinical conditions involving angiogenesis. The expression of IL-6, a multi-functional cytokine is elevated in tissues that undergo active angiogenesis, but it does not induce proliferation of endothelial cells. IL-6 has the ability to induce angiogenesis indirectly by the expression of VEGF [26] and possibly by increasing endothelial permeability [27].

Certain transcription factors like NF-Kappa B are known to activate synergistically transcription of cytokines such as IL-6 and chemokines IL-8 and MCP-1 [28], [29], [30]. Further, transcriptional activation of VEGF by IL-6 via STAT-3 pathway and transactivation of VEGF-R2 by IL-8 are associated with vascular permeability [31], [32]. Thus, IL-6, present in significant amount in vitreous of PDR and ED patients, may be able to function as indirect inducer of tube formation *in vitro*. Whether a similar IL-6 induced VEGF expression occurs in the endothelial cells in the tubulogenesis assay needs further studies.

RZB is a high affinity recombinant Fab which binds to the receptor–binding site of all biologically active forms of VEGF-A, thus preventing the activation of two related receptor tyrosine kinase, VEGFR-1 and VEGFR-2. Consequently, the endothelial cell proliferation, migration, permeability and vascular assembly are inhibited by RZB [33], [34], [35]. In this context our study was designed to evaluate the ability of RZB and anti-IL-6 antibodies to inhibit the angiogenic ability of PDR and ED vitreous. A marked reduction in vascular tube formation was observed when PDR vitreous was treated with RZB although this was not observed in all the samples. For example among the two PDR vitreous samples, though both contained low levels of VEGF, tube formation was significantly reduced by RZB in PDR-53 but not in PDR-49 and this difference may possibly be due to the presence of low levels of IL-6 and MCP-1 in the former but high levels of these cytokines in the latter. In general our results can be correlated with clinical studies wherein up to 38.6% of PDR patients did not respond to VEGF therapy [36].

In our context with IL-6 neutralizing antibody we observed tube length reduction in PDR; however, the reduction was not significant (Table-1, Fig. 5E). These samples contained high levels of MCP-1 and varying concentrations of other cytokines. In general, the present study suggests the involvement of inflammatory cytokines in addition to VEGF in inducing tube formation. Further studies are required to evaluate the inter-relationship between the expression of inflammatory cytokines and VEGF as well as their synergistic activity at the molecular level.

In conclusion, this is the first study to evaluate the angiogenic potential of vitreous from PDR and ED patients, demonstrating that VEGF present in ED vitreous is involved in inducing the vascular endothelial cell migration and assembly. Further, the importance of proinflammatory factors in addition to VEGF in retinal neovascularization is well-indicated. It is important to note that in patients, vitreous levels of growth factors and cytokines may not necessarily be due to the pathophysiology of the respective disease entity, but from intravitreal blood and associated cell sources such as thrombocytes. Nevertheless, in this study patient numbers are limited since the majority of vitreous samples we obtained needed to be excluded from the study due to blood contamination. Nevertheless, the study forms a basis for extending patient numbers and further investigating the cytokine profile in PDR and ED and how this influences key function endpoints such as pathological angiogenesis. Therefore, the current data is a useful platform for extending the investigation by using several functional endothelial cell migration, proliferation, and permeability assays [37], [38] to elucidate the importance of various factors (IL-8,MCP-1) and their interaction in neovascularization, by using the patients' vitreous and a combination of neutralizing antibodies.
